# Diet as a Risk Factor for Pneumococcal Carriage and Otitis Media: A Cross-Sectional Study among Children in Day Care Centers

**DOI:** 10.1371/journal.pone.0090585

**Published:** 2014-03-05

**Authors:** Terhi Tapiainen, Niko Paalanne, Tuula Arkkola, Marjo Renko, Tytti Pokka, Tarja Kaijalainen, Matti Uhari

**Affiliations:** 1 Department of Pediatrics, Oulu University Hospital, Oulu, Finland; 2 Department of Pediatrics, University of Oulu, Oulu, Finland; 3 National Institute for Health and Welfare, Oulu, Finland; Rockefeller University, United States of America

## Abstract

**Background:**

Pharyngeal bacteria are exposed to different sugar conditions depending on the diet of the child. We hypothesized that dietary factors such as daily intake of carbohydrates could be associated with pneumococcal carriage and the occurrence of otitis media in children.

**Methods:**

Our study design was a cross-sectional study among 1006 children attending child day care centers. Parents filled in a food frequency questionnaire. Oropharyngeal swabs were collected from each child. The primary outcome was the occurrence of pneumococcal carriage and the secondary outcome the number of acute otitis media episodes during life. Principal component analysis was used to group dietary intake into nine factors. The models were adjusted for age, gender of the child and educational level of the mother.

**Results:**

The dietary factor which included high consumption of sweet pastries and jam was associated with an increased risk of pneumococcal carriage (OR 1.17, 95% CI 1.01 to 1.36, P-value 0.04). The factor including frequent consumption of fruit and berries was associated with a decreased risk of acute otitis (regression coefficient −0.51, 95% CI −0.98 to −0.03, P = 0.04). A high intake of consumption of sweets and snacks (OR 1.36, 95% CI 1.03 to 1.80, P = 0.03) was associated with an increased risk of caries.

**Conclusions:**

Diet was associated with a risk of pneumococcal carriage and the occurrence of otitis media. Diet may thus be a modifiable risk factor for the occurrence of acute otitis media.

## Introduction

A significant proportion of children carry *Streptococcus pneumoniae* asymptomatically [1]. Pneumococcal carriage is more common among children attending day care centers than in other family members, and in many cases children attending day care introduce pneumococci into their family [2]. Nasopharyngeal carriage of *S. pneumoniae* is more common among children with respiratory infection, but carriage does not automatically lead to infection, and difference in its occurrence between healthy and symptomatic children is small [1]. *S. pneumoniae* is the most commonly reported bacterial cause of acute otitis media (AOM), accounting for 28 to 55 percent of cases [3].

There are only a few studies available on the effect of dietary habits on the risk of common pediatric infections in developed countries. Dietary habits, especially concerning increased consumption of sucrose, have been linked with dental caries [4], the consumption of berry juices has been associated with a decreased risk of urinary tract infections in children and women [5]–[7], and regular use of xylitol reduces the risk of otitis media in children [8]. In adults, poor glycemic control has been associated with an increased risk of pneumococcal pneumonia among patients with diabetes, while among non-diabetic patients hyperglycemia at the time of a pneumococcal pneumonia episode is associated with increased severity and mortality [9].

Pharyngeal bacteria are exposed to different sugar conditions depending on the diet of the child. Our hypothesis was that the diet of children, including features such as a high intake of sucrose, might affect the colonization by common otopathogens and could thus increase the risk of AOM.

We therefore designed a cross-sectional study among day-care children in order to find possible associations between pneumococcal carriage and diet. In addition, we recorded the AOM history of each child together with known risk factors for AOM.

## Subjects and Methods

### Population

Children were recruited from 57 child day care centers in the city of Oulu, Finland (Table 1) and data were collected between May 2010 and Nov 2010. The study physicians gave an educational lecture to the parents of the children at these day care centers prior to the enrolment procedure, informing them that the intention was to elucidate the association between pneumococcal carriage and dietary factors in general. The specific hypothesis concerning the possible effect of a high intake of sucrose on AOM morbidity was not explained.

**Table 1:** Characteristics of the children (n = 1006).

Characteristic	Subcategory	Number of children (%)
Age, years mean (SD)		4.3 (1.7)
Gender	Male	515 (51)
	Female	491 (49)
Breastfeeding at least 6 months		733 (73)
Weight^1^	Normal weight	742 (83)
	Overweight	124 (14)
	Obese	29 (3)
Pneumococcal vaccination^2^		59 (6)
Mothers’ educational level	Elementary school	24 (3)
	Senior high school	65 (7)
	Vocational school	164 (17)
	Polytechnic	362 (36)
	University	369 (37)
Smoking	Parental smoking^3^	239 (24)
	Maternal smoking	110 (11)
Dental caries diagnosed by a dentist		64 (6)
Asthma diagnosed by a physician		68 (7)
Previous history of AOM^4^	0 episode	175 (18)
	1–5 episodes	488 (49)
	>5 episodes	292 (29)
Adenoidectomy performed		117 (12)
Current tympanostomy tubes		20 (2)

1 Overweight means ISO-BMI between 25 and 30, obesity means ISO-BMI more than 30.

2 41 of the children received a 10-valent vaccination, 13 received a 13-valent vaccination, 5 received a 23-valent vaccination, and the vaccination data for 26 children were unknown. Pneumococcal 10-valent vaccination is now part of the Finnish national immunization programme for children born after 1st June 2010.

3 Any smoking by the mother or father at the time of the survey.

4 Number of AOM episodes reported by the parents.

**Figure 1:**
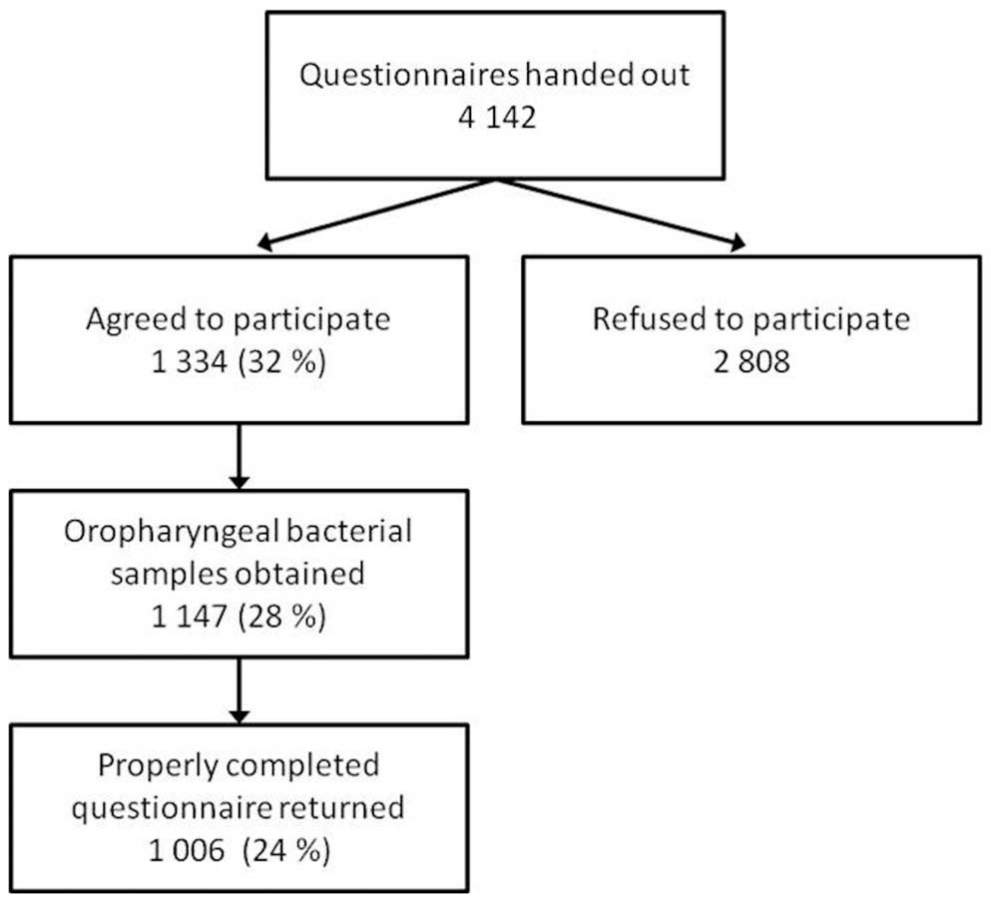
Study design.

Information was handed out to the parents of 4142 children via the day care centers, and the parents of 1334 children enlisted and filled in the questionnaires. Out of this number, 1147 children were present when the nurse visited at their day care center. Thus we were able to collect a oropharyngeal bacterial sample from 1147 participating children (28% of those whose parents were originally contacted), a figure that was reduced to 1006/4142 (24.3%) when accepting only those children who had a properly completed dietary habit questionnaire for inclusion in the analysis (Figure 1).

In the food frequency questionnaire (FFQ) the parents were asked to record the frequency of child’s consumption of various foods and drinks during the past month on a seven-point scale ranging from never to twice a day or more. A total of 65 food items was enquired about in this way, and open spaces for additional foods and drinks were provided and the information given on these was coded by the nutritionist and included in the eventual data. The dietary data were further aggregated to form 25 food groups prior to the statistical analysis, the grouping scheme being based on culinary use and nutrient profiles. The intake of vitamin D from supplements was calculated by combining the intake from vitamin D and multivitamin supplements. A high proportion of children regularly had xylitol chewing gum or xylitol lozenges, 86% of the children received xylitol every day at the day care centers and 94% were given xylitol at least once a week at home and 51% daily. 50% of the children received additional Vitamin D daily, whereas 33% did not have any additional Vitamin D. Xylitol and vitamin D were not included in the food group analysis.

Another questionnaire was used to collect data on previous medical history. The total number of previous AOM episodes diagnosed by a physician, the date of the first and last episodes and the dates of any surgery for recurrent AOM (tympanostomy tube placement and/or adenoidectomy) were collected, as also was background information relevant to known risk factors for AOM (age, gender, number of siblings, parents’ educational level, exposure to tobacco smoke, breast feeding history and vaccination history). We similarly asked the parents about the child’s history of dental caries, since its risk is known to be affected by dietary factors [10], and about the child’s weight and height at the latest check-up at a clinic. Only 6% of the participants had received pneumococcal conjugate vaccine, since this was not included in the national immunization program at that time. Pneumococcal conjugate immunization has now been started in Finland for all children born on 1st June 2010 or later, but none of the present subjects had been born after that date.

### Ethics Statement

The study was approved by the institutional ethics review board of Oulu University Hospital. The participants took part on a voluntary basis and their parents gave their written informed consent.

### Bacteriological Methods

The pharyngeal samples taken by a trained nurse at the day care centers were analyzed for the presence of *S. pneumoniae*, *Haemophilus influenzae*, *Moraxella catarrhalis*, *Staphylococcus aureus* and *Streptococcus pyogenes*. The sample was taken with a calcium alginate swab via the oral cavity and from a point as close to nasopharynx as possible and immediately immersed in a tube containing 1 ml of STGG (skim milk, tryptone, glucose, glycerol) medium [11]. The tube with the swab was immediately vortexed and stored in a portable cooler box at the refrigerator temperature (+4°C–+8°C) for not more than 8 hours. The samples were then frozen at ≤ −65°C on the same day in the bacteriological laboratory (National Institute for Health and Welfare, Oulu, Finland). For the analysis 10 µl of each sample was later cultured on 5% sheep blood agar, sheep blood agar with gentamicin (5 µg/ml) and chocolate agar plates. The plates were incubated in 5% CO_2_ at +35°C–+37°C for 18 to 24 hours and colonies suspected of being *S. pneumoniae*, *H. influenzae*, *M. catarrhalis*, *S. aureus* and *S. pyogenes* were identified using generally accepted methods as described earlier [3]. The number of pathogenic bacterial colonies and the normal nasopharyngeal flora were estimated and recorded as negative (no colonies), +(1–10 colonies), ++(11–100 colonies) and+++(>100 colonies). The oropharyngeal swabs were collected between May and June and between September and November in 2010 and all the samples were cultured in the spring of 2012.

### Outcome, Exposure and Sample Size

The primary outcome was the occurrence of pneumococcal carriage and the secondary outcome the number of acute otitis media episodes during life. The exposure was different dietary habits according to dietary factors analyzed using principal component analysis (PCA). Before the study, we estimated the prevalence of pneumococcal carriage to be 30% [1], [2], and considered an odds ratio of 1.5 for pneumococcal carriage to be clinically important in the comparison of dietary habits. Using a 0.05 type I error (P-value) and a power of 80%, a sample size of 424 children per dietary group (e.g. those with a higher than average intake of sucrose) was required, i.e. altogether 850 children with bacterial samples and properly completed questionnaires. Thus we aimed to recruit at least 1000 children for the study.

### Statistical Methods

In order to determine how pneumococcal carriage and the number of AOM episodes relate to dietary habits, principal component analysis with varimax rotation was used to extract nine factors from the nutrition variables (Table 2). On the basis of preliminary analysis certain items with a low communality value (milk, sour milk, and natural yoghurt) were excluded from the final PCA analysis, in which all factors with an eigenvalue greater than one were retained for further analysis. The factor scores were computed using the least squares regression approach and taken as predictive variables in the subsequent analysis. Multivariate logistic regression and general linear model (GLM) analyses were performed with the factor scores as independent variables and were adjusted for the age and gender of the child and the socioeconomic status of the mother. The effects of the nutrition variables on pneumococcal carriage and the number of otitis media episodes are presented as odds ratios (OR) and regression coefficients. IBM SPSS Statistics for Windows (version 20.0.0, SPSS Inc., Chicago, IL, USA) was used in the analyses.

**Table 2:** Dietary groups revealed by the factorial analysis^1^.

Group	Predominant food items
1 Sweets	Sweet milk products, sweet drinks, light drinks, sweets and chocolate, salty snacks, fast food, sausages
2 Meat	Meat, poultry, fish, sausages, eggs
3 Bread	Bread, cheese, sausages, vegetable margarine and oil
4 Fruit	Fruit and berries, vegetables, light drinks, jam and honey
5 Cereal	Sweet cereals, breakfast cereals, sweet milk products, sweet drinks
6 Biscuits	Sweet pastries and biscuits, jam and honey, sweets and chocolate, sweet drinks
7 Butter	Butter
8 Home food	Potatoes, porridge, sweet berry compotes, meat, light drinks
9 Pasta	Pasta and rice, poultry, sweet berry compotes, sugary berry juice

1 The dietary groups represent factors revealed by principal components analysis (PCA) with varimax rotation among 1006 children. A total of 25 food items were used in the analysis.

## Results

The children had commonly been consuming foods that contained high amounts of added sugar, the most frequent being flavored yoghurt, sweetened fruit drinks and sweet pastries. About 30% of the children ate sugary yoghurt daily and 10% drank sweetened drinks at least twice a week. Fruit and berries were consumed daily by 20% of the children. About 3% of the children were obese (Table 1).

A total of 246 children (24.5%) had *S. pneumoniae* in their sample, the risk being greatest at a younger age (OR 0.80 per one year 95% CI [confidence interval] 0.73 to 0.89 P<0.01). After adjustment of the analysis for the child’s age and gender and the mother’s educational level the factor involving high consumption of sweet pastries and jams (Group 6) was associated with an increased risk of pneumococcal carriage (OR 1.17, 95% CI 1.01 to 1.36, P-value 0.04) (Table 3).

**Table 3:** Risks of pneumococcal carriage and AOM episodes attached to the food groups^1^.

	Pneumococcal carriage			AOM episodes		
Food group	OR	95% CI for OR	P-value	β^2^	95% CI for β	P-value
1 Sweets	0.98	0.84 to 1.15	0.81	0.08	−0.42 to 0.58	0.76
2 Meat	0.93	0.80 to 1.09	0.36	0.08	−0.39 to 0.56	0.73
3 Bread	1.02	0.88 to 1.18	0.83	0.17	−0.31 to 0.65	0.48
4 Fruit	1.10	0.95 to 1.28	0.23	−0.51	−0.98 to −0.03	0.04
5 Cereal	0.99	0.85 to 1.17	0.93	0.39	−0.09 to 0.87	0.11
6 Biscuits	1.17	1.01 to 1.36	0.04	−0.29	−0.77 to 0.19	0.24
7 Butter	1.02	0.88 to 1.19	0.79	−0.23	−0.70 to 0.23	0.33
8 Home food	0.87	0.74 to 1.02	0.08	0.28	−0.21 to 0.77	0.28
9 Pasta	0.98	0.84 to 1.15	0.83	−0.33	−0.81 to 0.15	0.17

Results of the multivariate logistic regression analysis and general linear model (GLM) analysis.

1 Adjusted for age, gender and maternal educational level.

2 Regression coefficient, values below zero indicate decreased risk and those above zero increased risk.

The majority of the children had had at least one AOM episode and almost 30% had suffered from more than five (Table 1). The factor that included frequent consumption of fruit and berries (Group 4) was associated with a decreased risk of acute otitis (regression coefficient −0.51, 95% CI −0.98 to −0.03, P = 0.04) (Table 3).

The factor implying a high consumption of fast food, sweets and salty snacks (Group 1) showed a significant association with the increased risk of dental caries (OR 1.36, 95% CI 1.03 to 1.80, P = 0.03), as also did a high intake of sugared cereals and sugary dairy products (Group 5) (OR 1.36, 95% CI 1.09 to 1.71, P<0.01). There were also statistically significant associations between other bacteria and dietary habits (Table 4).

**Table 4:** Risks of carriage of *Haemophilus influenzae* (91/1006, 9%), *Moraxella catarrhalis* (179/1006, 18%) and *Staphylococcus aureus* (261/1006, 26%) attached to the food groups^1^.

	*H. influenzae*			*M. catarrhalis*			*S. aureus*		
Food group	OR	95% CI	P-value	OR	95% CI	P-value	OR	95% CI	P-value
1 Sweets	1.15	0.92 to 1.41	0.21	0.92	0.77 to 1.10	0.36	1.10	0.94 to 1.27	0.23
2 Meat	0.89	0.70 to 1.12	0.32	0.97	0.82 to 1.15	0.73	0.89	0.76 to 1.04	0.14
3 Bread	0.99	0.79 to 1.23	0.89	1.05	0.88 to 1.24	0.61	0.92	0.79 to 1.06	0.24
4 Fruit	1.13	0.92 to 1.39	0.24	1.15	0.97 to 1.36	0.11	0.83	0.72 to 0.96	0.01
5 Cereal	1.03	0.82 to 1.28	0.82	0.91	0.75 to 1.10	0.31	0.97	0.84 to 1.12	0.67
6 Biscuits	1.09	0.89 to 1.33	0.38	1.22	1.03 to 1.43	0.02	1.02	0.89 to 1.18	0.75
7 Butter	1.01	0.81 to 1.26	0.95	0.83	0.69 to 0.99	0.04	0.99	0.87 to 1.16	0.99
8 Home food	1.16	0.94 to 1.44	0.17	0.99	0.84 to 1.18	0.93	1.08	0.93 to 1.26	0.31
9 Pasta	0.79	0.64 to 0.98	0.04	0.95	0.80 to 1.13	0.57	0.95	0.82 to 1.10	0.46

Results of the multivariate logistic regression analysis.

1 Adjusted for age, gender and maternal education level.

## Discussion

We found that the children’s diet did show an association with pneumococcal carriage and the occurrence of otitis media. Known modifiable risk factors for AOM are few in number. The risk has been reported to be increased by maternal smoking and by the use of a pacifier [12], [13], and the evidence for a protective effect of breastfeeding is convincing [12], [14]. All the present children were attending day care, which is in itself a well-documented risk factor for AOM [12]. Our results suggest that diet could be one modifiable risk factor when seeking to reduce AOM morbidity.

The dietary factors studied here also showed a marked association with the risk of dental caries. The association between diet, especially sucrose intake, and dental caries is well documented [10]. The fact that our result in this respect is in accordance with previous findings, supports the validity of our factorial analysis. The virulence of both *S. pneumoniae* and *S. mutans*, the main pathogen responsible for dental caries, is affected by environmental sugar conditions, as the oral streptococci are adapted to utilizing the rapidly fluctuating supplies of sugar in the oral cavity [15], [16]. Both dental caries and AOM have been shown to diminish if a non-utilizable sugar alcohol, xylitol, is consumed regularly in the diet [8], [17].

Our results show that an increased intake of fruit and berries could be protective against AOM. The consumption of fruit and berries is generally linked with cardiovascular health and a decreased risk of cancer [18], while the prevention of urinary tract infections by the regular consumption of berry products has been attributed to certain specific flavonoids which are potent inhibitors of the adhesion of coliform bacteria to human cells [5]. Furthermore, the flavonoids and proanthocyanidins in cranberry extracts seem to reduce the formation of biofilms and the acidogenicity of *S. mutans* [19], [20].

The factor that included the frequent consumption of fruit and berries also included low levels of sweets, sugared cereals and sweet pastries, all of which were associated with an increased risk of pneumococcal carriage. As the subjects were very similar in their total food intake, an increased intake of fruit and berries would lead to a decrease in other items. It is therefore possible that in addition to the protective effect of fruit and berries, the results may have been affected by a decrease in the consumption of some food item that entails a risk of pneumococcal carriage.

Our *a priori* hypothesis was that dietary factors could affect pneumococcal carriage. After these analyses, we also tested the association of other nasopharyngeal bacteria and diet. High consumption of fruit and berries associated with decreased carriage of *S. aureus.* In addition, the food group including high intake of biscuits and other sweet pastries associated with carriage of *M. catarrhalis.* The carriage rates of otopathogens were lower in our study than in some earlier reports [21]. We used oropharyngeal sampling since it was easier to do in day care centers, even though the oropharyngeal sampling may result in lower detection rates of otopathogens than nasopharyngeal sampling [22], [23].

The rather low participation rate (24%) may hamper the generalization of our results. The maternal educational level in this population was high, as more than 70% of the mothers had a polytechnic or university degree whereas the average proportion of Finnish women under the age of 35 years who have an education at this level is 42% [24]. It should be noted, too, that previous Finnish population studies have reported that a higher parental educational level is associated with a healthier diet for the children [25]. 10-valent pneumococcal conjugate vaccination was included in the Finnish national immunization program in 2010. Our series was recruited prior to the pneumococcal conjugate vaccination era in this country, and consequently only six percent of subjects had received this vaccination. Pneumococcal conjugate vaccine has had only a minor effect on overall pneumococcal carriage and the occurrence of AOM, as the vaccine-related serotypes of *S. pneumoniae* have been replaced by non-vaccine-related serotypes [26], [27].

The consumption of fresh vegetables, fruit and berries, fish and fats derived from vegetable oils is generally said to be too low among children in many countries, whereas the consumption of foods containing sucrose and salt is higher than that recommended [25], [28]. The overall quality of a child’s diet starts to decline after the age of one year, when the child begins to take part in family meals [25]. A healthy diet can protect children against numerous health problems including obesity and dental disorders. In this respect the present results provide one more reason for following such recommendations for healthy nutrition in childhood.

### Conclusions

The risk of pneumococcal carriage and the occurrence of AOM were associated with the children’s diet. Diet may thus be a novel modifiable risk factor for acute otitis media.
